# Anthelmintic control failure and associated risk factors reported by farmers in communal sheep farming, Oliver Tambo District, Eastern Cape, South Africa

**DOI:** 10.1002/vro2.70022

**Published:** 2025-11-14

**Authors:** Songezo Mavundela, William Diymba Dzemo, Oriel Thekisoe

**Affiliations:** ^1^ Department of Biological and Environmental Sciences Walter Sisulu University Mthatha South Africa; ^2^ Unit for Environmental Sciences and Management North‐West University Potchefstroom South Africa

**Keywords:** anthelmintic, communal farming, control failure, sheep production

## Abstract

**Background:**

Helminth control on communal farms of South Africa, primarily relies on anthelmintic drugs, administered by farmers without veterinary supervision. This study investigated risk factors associated with farmer‐reported anthelmintic control failure on communal sheep farms in Oliver Tambo District of the Eastern Cape of South Africa.

**Methods:**

A semi‐structured questionnaire capturing farm characteristics, helminth control practices and treatment failure was administered to 107 farmers between January and August 2024.

**Results:**

Most of the farmers encountered were older males (56%) aged over 65 years. Helminth eggs were identified in sheep faecal samples, revealing four genera: *Haemonchus* spp. (100%), *Strongyloides* spp. (67%), *Moniezia* spp. (61%) and *Trichuris* spp. (17%). A total of 15 anthelmintic drug brands were recorded. The most commonly used compounds (46%) were Valbantel, Maxicare and Prodose Orange, all co‐formulations of benzimidazoles and salicyanilides. Anthelmintic control failure was reported by 62% of farmers. The predominant sheep crossbreed raised on the farm (Dorper‒Merino), the use of 5‐ or 10‐mL syringes for anthelmintic drug administration, and the absence of sheep weight estimation were statistically associated with farmer‐reported anthelmintic control failure.

**Conclusion:**

These findings underscore the need for improved helminth management strategies to enhance the effectiveness of anthelmintic treatments in communal farming systems.

## INTRODUCTION

Livestock production in South Africa accounts for approximately 49% of the country's national agricultural output.^1^ The Eastern Cape of South Africa leads in livestock productivity, contributing substantial proportions to the national totals, with 24% cattle, 38% goats and 29% sheep.^2^ The Oliver Rignald Tambo District Municipality (ORTDM) has the largest sheep population in Eastern Cape, estimated at approximately 1.2 million, with more than half raised in communal areas.^3^ In communal farming areas, farmers collectively utilise designated grazing lands for various livestock species, including cattle, goats, sheep and horses.^4^, ^5^ Sheep rearing in communal areas of the ORTDM plays a critical role in the livelihoods of resource‐poor farmers, providing protein and financial needs through the sales of milk, wool and hides^6^ while also contributing to farmers’ social status.^7^ However, sheep productivity on communal farms is hindered by limited access to effective veterinary services,^8^, ^9^ diseases, poor management practices and parasitism.^6^, ^9^ Gastrointestinal parasitism, particularly by helminth infections, poses a significant health challenge for small ruminants in Eastern Cape.^10^ *Haemonchus contortus*, *Trichostrongylus* spp. and *Strongyloides* spp. are among the most prevalent helminths in the province.^10^ Heavy helminth infections in sheep can significantly reduce productivity, causing weight loss, reduced immunity, lower meat and wool quality, reduced fertility, anaemia, lower milk production, and increased morbidity and mortality rates.^8^ Helminth control in communally reared sheep involves the use of anthelminthic drugs, which are purchased and administered by farmers without veterinary supervision.^11^ The extensive and indiscriminate use of anthelmintic drugs has led to the development of anthelmintic resistance (AR).^8^, ^9^, ^12^, ^13^ Helminth parasitism, their control with anthelmintic drugs, and the issue of AR have serious economic repercussions for the global sheep production industry.^14^, ^15^ In Tropical Africa, annual losses due to the costs of treating helminth infections in sheep have been estimated at USD 77.9 million in Nigeria,^16^ USD 3.7 million in Ethiopia,^17^ USD 26 million in Kenya^18^ and USD 45 million in South Africa.^19^ The development of AR is strongly driven by poor helminth control practices.^20^ Several helminth control practices have been linked to an increased likelihood of AR and, consequently, anthelmintic control failure. These include high treatment frequency, under‐dosing, lack of monitoring for drug efficacy, absence of drench and shift approach, absence of rotation of anthelmintic classes, sheep breed and blanket treatment.^21^, ^22^, ^23^, ^24^ Mphahlele et al.^8^ found that the lack of accurate weight to calculate dose and limited farmer experience were associated with the occurrence of AR in sheep of resource‐poor farms in Limpopo, South Africa. In Eastern Cape, current research studies have focused on various aspects of sheep production and helminth control, including farmers' perceptions and rankings of the most important constraints to sheep production,^6^ prevalence and seasonal variation of gastrointestinal nematodes (GINs),^10^ the identification and collection of plant species used to control GINs in livestock^25^ and communal sheep farmers' knowledge and attitudes towards GINs.^26^ However, an assessment of helminth control practices contributing to anthelmintic failure on resource‐poor communal sheep farms in the Eastern Cape has not yet been conducted. Understanding these practices is crucial for the development of evidence‐based strategies to mitigate anthelmintic control failure and ensure the sustainable use of available anthelmintics. Ultimately, this will lead to healthier animals, improved productivity, increased income for small‐scale farmers and enhanced food security.

This study attempted to investigate risk factors contributing to the farmer‐reported anthelmintic control failure in sheep on communal farms in ORTDM of the Eastern Cape of South Africa.

## MATERIAL AND METHODS

### Study area and design

**FIGURE 1:**
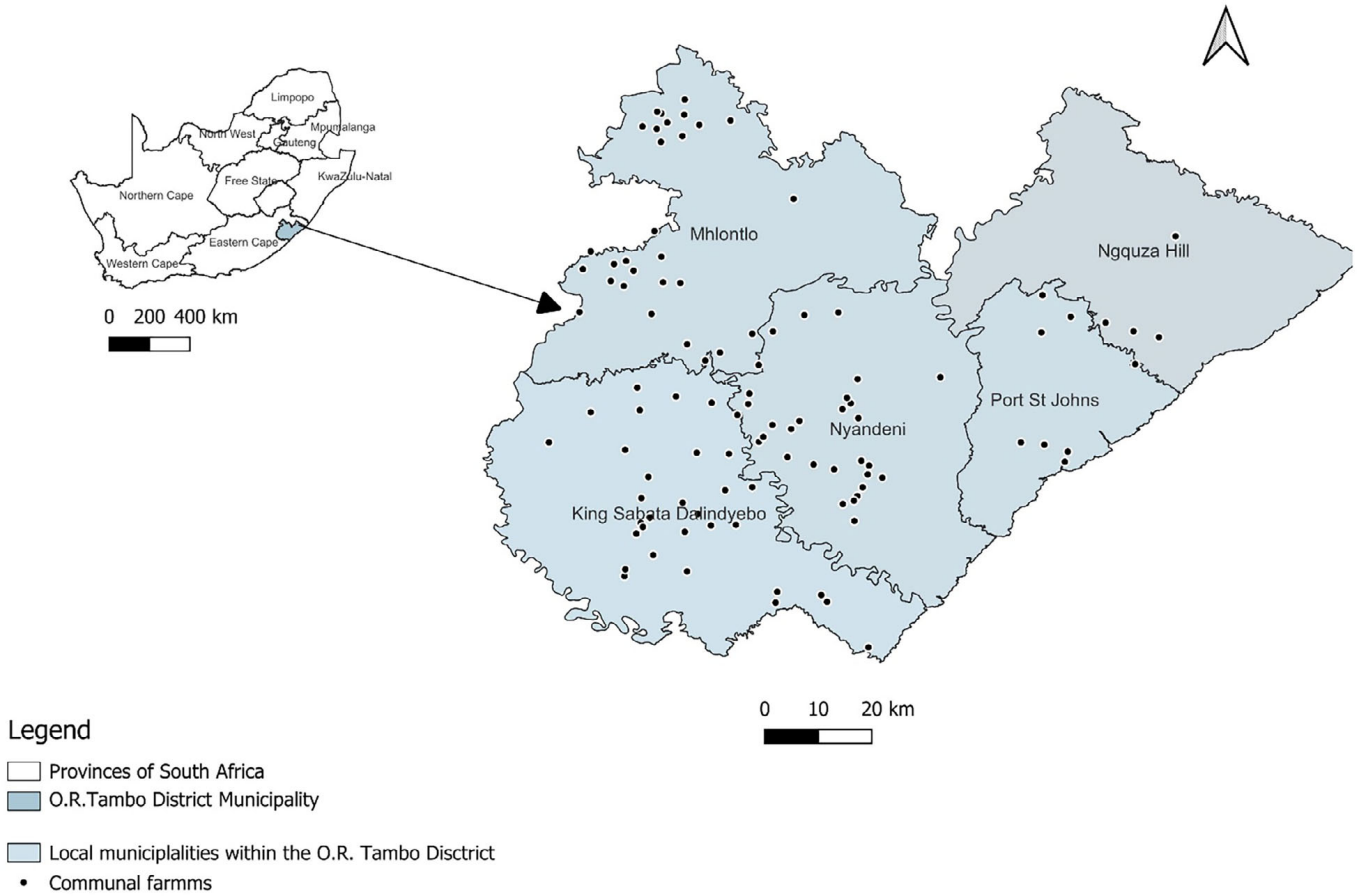
Map of Oliver Reginald Tambo District Municipality showing the communal farms included in the survey study.

The ORTDM is located on the eastern coastal portion of the Eastern Cape, South Africa^27^, ^28^ (Figure 1). The ORTDM extends over a geographical area of 15, 947 km^2^ ^27^ and incorporates five local municipalities, namely, King Sabata Dalindyebo, Mhlontlo, Nyandeni, Port St Johns and Ngquza Hill. Most parts of the ORTDM experience annual rainfall exceeding 900 mm.^28^ Furthermore, the ORTDM experiences temperatures ranging from 16°C to 28°C and from 7°C to 20°C during the summer and winter seasons, respectively.^27^ The District has diverse habitats, including inland and coastal grasslands, Afromontane and coastal forests, valley thickets, thorny bushveld, as well as coastal and marine ecosystems.^27^, ^28^ The district has the largest number of communally reared livestock in South Africa, incorporating 631,674 cattle, 732,478 goats and 1,225,244 sheep.^3^ Farmers graze their livestock on both grassland‐only and grassland‐and‐forest areas.^28^ Communal areas consist of villages where the land is divided for residential use, farming and grazing. In these communal areas, livestock farmers collectively share allocated rangelands for the pasturing of different livestock including cattle, goats, sheep and horses.^4^, ^5^, ^28^ These farmers varied in their deworming practices, with some treating animals using different anthelmintics, while others did not administer treatments. Other agricultural activities practiced within the district include crop farming, forestry and aquaculture.^28^

A cross‐sectional survey study was designed to document the risk factors contributing to the development of anthelmintic control failure on communally reared sheep of the ORTDM. Prior to the commencement of the study, an authorisation letter and data on communal sheep farmers were obtained from the Office of the Oliver Rignald Tambo District Veterinary Services. The inclusion criteria for selecting participants were as follows:

1. Communal sheep farmers who kept more than 40 sheep.
2. Farmers who had been rearing sheep for over a year.
3. Farmers who were using anthelmintic drugs for the control of helminths.

Eligible farmers meeting these criteria were purposively recruited from all five local municipalities within the ORTDM (King Sabata Dalindyebo, Nyandeni, Port St Johns, Ngquza Hill, and Mhlontlo).

### Survey instrument and helminth egg detection

A semi‐structured questionnaire was developed and administered from January to August 2024 to gather data on sheep farm characteristics, the presence of helminths, their effects and control approaches, anthelmintic drug usage and application practices, as well as reported anthelmintic control failure on communal farms. A farm was deemed to have anthelmintic control failure when helminth eggs detected in faecal samples on days 7 and 14 post‐treatment had an egg per gram 200 or more, and the farmer reported treatment failure, the following clinical signs after anthelmintic administration: bottle jaw, rough and poor‐quality fleece, and persistent diarrhoea. The survey tool was tested for validity and clarity to ensure that it was free of ambiguity. The pilot test was conducted by seven randomly selected sheep farmers and three animal health technicians (AHTs). The contact details and farm locations of the recruited communal sheep farmers were obtained with the help of AHTs and community animal health workers. The administration of questionnaires was conducted telephonically to farmers who could not avail themselves on the farm and whose farms were not easily accessible. The survey administrator conducted face‐to‐face interviews with farmers who were present on the farm and whose farms were easily accessible. Interviews were conducted in the local isiXhosa language, as most communal farmers were unfamiliar with some of the technical terminologies used in the questionnaire. Prior to the administration of the questionnaire, the purpose of the study was explained to the selected respondents, and informed consent was obtained for the interview.

At the farms visited, faecal samples were collected from five randomly selected lambs that had been previously treated with an anthelmintic by the farmer (Table 1). The interval between anthelmintic administration and faecal collection was 7 and 14 days. The faecal samples were pooled,^29^ using 2 g of faeces per animal, and thoroughly homogenised. Approximately 2 g of the pooled faecal samples were weighed and mixed with about 28 mL of sodium chloride flotation solution, which was prepared to a specific gravity of approximately 1.20. The McMaster technique, described in Zajac and Conboy,^30^ was used to identify the presence of helminth eggs under a compound microscope (Zeiss Upright Microscope Primo Star). Morphological identification of the helminth eggs encountered was performed as described by Zajac and Conboy.^30^

**TABLE 1:** Anthelmintic drugs administered orally on communal sheep farms in the Oliver Reginald Tambo District, South Africa.

Trade name	Active ingredient(s) (anthelmintic class/es)	Number of farms administering the anthelmintic
Valbazen	Albendazole 1.9% (m/v) and Closantel sodium 3% (m/v) (BZ + SLD)	30
Prodose Orange	Albendazole 1.9% (m/v) + Closantel sodium 3.94% (m/v) (BZ + SLD)	10
Valbazen	Albendazole 1.9% (m/v) (BZ)	9
Endo + Lint	Levamisole HCI 3.75% (m/v) + Praziquantel 1.88% (m/v) (IMD + ISN)	5
Prodose Red	Levamisole hydrochloride 2.56% (m/v) (IMD)	4
Lintex‐L	Niclosamide 20% (m/v) (SLD)	5
Ecomectin	Ivermectin 0.08% (m/v) (ML)	5
Virbamax First Drench	Abamectin 0.08 + % (m/v) + Praziquantel 1.50% (m/v) (ML + ISN)	3
Nemarox	Levamisole 2.5% (m/v) + Rafoxanide 2.5% (m/v) (IMD + ISN)	2
Flukazole C	Triclabendazole 12% (m/v) + Oxfendazole 4.53% (m/v) (BZ)	5

Abbreviations: BZ, benzimidazoles; IMD, imidazothiazoles; ISN, isoquinoline; ML, macrocyclic lactones; SLD, salicylanilides.

### Data analysis

The data obtained from the questionnaires were summarised, manually coded, and entered into Microsoft Excel for Microsoft 365. Counts, frequencies and percentages were generated and analysed in Statistical Package for the Social Sciences (version 29.0, IBM). A helminth control practice was considered to be statistically associated with the reported anthelmintic control failure if its corresponding *p*‐value of the Pearson's chi‐square statistic was 0.05 or less.

## RESULTS

A total of 180 farmers were contacted, and 107 participated in the cross‐sectional study, of whom 29 were interviewed telephonically and the remaining (*n* = 78) were visited for face‐to‐face interviews (Table 2). They relied on communal livestock rearing as their primary source of livelihood. The most reared sheep breed within the ORTDM was Dorper‒Merino crossbreed (96%) with 75% of the farms having flock sizes greater than 100. The majority (79%) of the farmers had over 10 years of sheep farming experience. They raised sheep primarily for meat production (100%), ceremonial purposes (95%) and monetary exchange (94%). Their source of livestock was from fellow farmers within the community or village (57%). Communally reared sheep primarily graze on grassland pastures (74%) and were often kept alongside other ruminants, including cattle, goats, horses and donkeys. There is a high level (99%) of interaction between livestock species from different community members (Table 2).

**TABLE 2:** Demographics and characteristics of the studied communally reared sheep farms of the Oliver Reginald Tambo District, South Africa.

Query	Response category	Number of responses (*n* = 107)	Percentage (%)
Number of respondents per local municipality	King Sabata Dalindyebo	37	35
	Mhlontlo	31	29
	Nyandeni	27	25
	Port St Johns	8	7
	Ingquza hill	4	4
Gender	Male	101	94
	Female	6	6
Age group	18‒35 years	7	7
	36‒55 years	40	37
	>65 years	60	56
Level of education	Below matric	75	70
	Post matric	32	30
Employment status	Unemployed	103	96
	Formal employment	4	4
Sheep crossbreed reared	Dohne‒Merino	9	8
	Dorper‒Merino	98	92
Number of sheep	>100	80	75
	<100	27	25
Farming experience (years)	0‒10	22	21
	>10	85	79
Purpose for rearing sheep^a^	Meat	107	100
	Ceremonial purposes (bride price, ancestral rituals)	102	95
	Selling for income	101	94
	Wool production	84	79
Source of sheep on the farm	Farmer within the community/village	61	57
	Farmer from another community/village	46	43
Pasture animals graze on	Grassland	79	74
	Grassy woodland	23	21
	Thornveld	5	5
Keeping other ruminants	Yes	96	90
	No	11	10
Sheep interacting with neighbour's livestock	Yes	106	99
	No	1	1

^a^ Farmers provided multiple responses to the query.

**FIGURE 2:**
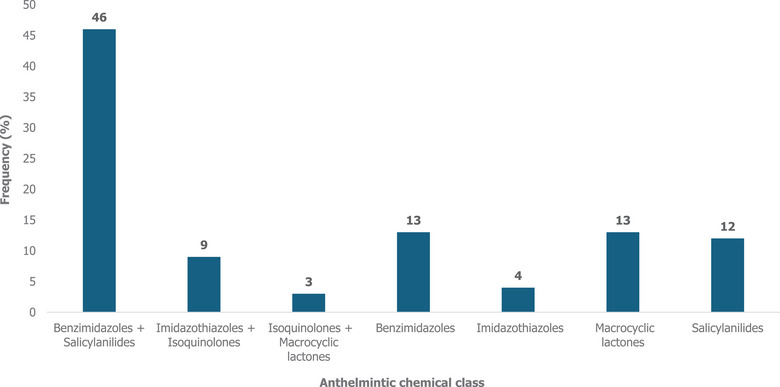
Single and combinations of anthelmintic chemical classes currently used on communally reared sheep farms in the Oliver Reginald Tambo District, South Africa.

Helminth eggs belonging to four distinct genera were detected from the faecal samples: *Haemonchus* spp. (100%), *Strongyloides* spp. (67%), *Moniezia* spp. (61%) and *Trichuris* spp. (17%) (Table 3 and Figure S1). The most reported clinical signs of helminth infection included weight loss (92%), diarrhoea (90%), rough and poor‐quality fleece (85%) and bottle jaw (83%). These clinical signs were more common during the summer season (57%). All the farmers treated sheep for worms using synthetic anthelmintic drugs, while a few others (4%) also supplemented these treatments with plant extracts. A total of 15 anthelmintic drug brands belonging to five anthelmintic chemicals were used by farmers (Table 4 and Figure 2). The most commonly used compounds (46%) were Valbantel (Zoetis) and Maxicare (Ceva Animal Health), both containing albendazole (1.9%, m/v) (benzimidazoles) and closantel sodium (3%, m/v) (salicyanilides), as well as Prodose Orange, which contains albendazole (1.9%, m/v) and closantel sodium (3.94%, m/v) (Table 4). Plant extracts of aloe vera (1%) and elephant's root (3%) were used to supplement anthelmintic drugs. Most farmers (99%) reported using the same anthelmintic drug for over a year.

**TABLE 3:** Helminth parasitism in sheep on communal farms of the Oliver Reginald Tambo District, South Africa.

Query	Response category	Number of responses	Percentage (%)
Helminth eggs detected (*n* = 78)^a^	Strongylid	78	100
	*Strongyloides* spp.	52	67
	*Moniezia* spp.	48	61
	*Trichuris* spp.	13	17
Farmer‐reported clinical signs of helminth parasitism^b^	Weight loss	98	92
	Diarrhoea	96	90
	Reduced appetite	73	68
	Rough and poor‐quality fleece	91	85
	Bottle jaw	89	83
	Constipation	72	67
Season associated with high worm infection rates	Summer	61	57
	Winter	11	10
	Autumn	10	9
	Spring	16	16
	Whole year	9	8
Do you administer worm treatment to your sheep?	Yes	107	100
	No	0	0
What do you use to treat worms?	Anthelmintic drugs only	103	96
	Anthelmintic drugs and plant extracts	4	4

^a^ Faecal samples collected from 78 of the 107 farms for detection of helminth eggs.

^b^ Farmers provided multiple responses to the query.

**TABLE 4:** Anthelmintic drugs currently used to control helminths on communally reared sheep farms in the Oliver Reginald Tambo District, South Africa.

Trade name	Active ingredient (anthelmintic drug class/es)	Number of responses (%) (*n* = 107)	Duration of use response (%)	
			<1 year	>1 year
Valbantel/Maxicare	Albendazole 1.9% (m/v) and Closantel sodium 3% (m/v) (BZ + SLD)	35 (33)	0	35 (33)
Prodose Yellow LA	Closantel 7.5% (m/v) (SLD)	3 (3)	0	3 (3)
Nemarox	Levamisole 2.5% (m/v) + Rafoxanide 2.5% (m/v) (IMD + ISN)	3 (3)	0	3 (3)
Flukazole C	Triclabendazole 12% (m/v) + Oxfendazole 4.53% (m/v) (BZ)	5 (5)	0	5 (5)
Virbamax First Drench	Abamectin 0.08 + % (m/v) + Praziquantel 1.50% (m/v) (ML + ISN)	3 (3)	1 (1)	2 (2)
Prodose Orange	Albendazole 1.9% (m/v) + Closantel sodium 3.94% (m/v) (BZ + SLD)	14 (13)	3 (3)	11 (10)
Ecomectin	Ivermectin 0.08% (m/v) (ML)	5 (6)	2 (2)	3 (3)
Endo + Lint/Eradiworm tape	Levamisole HCI 3.75% (m/v) + Praziquantel 1.88% (m/v) (IMD+ISN)	7 (7)	0	7 (7)
Ivermax	Ivermectin 1% (ML)	4 (4)	0	4 (4)
Lintex‐L	Niclosamide 20% (m/v) (SLD)	10 (9)	1 (1)	9 (8)
Ivomec	Ivermectin 0.08% (m/v) (ML)	4 (4)	0	4 (4)
Dectomax	Doramectin 1% (m/v) (ML)	1 (1)	0	1 (1)
Valbazen	Albendazole 1.9% (m/v) (BZ)	9 (8)	0	9 (8)
Prodose Red	Levamisole hydrochloride 2.56% (m/v) (IMD)	4 (3)	0	4 (3)
Aloe vera (Ikhala)	Plant extract	1 (1)		
Elephant's root (Intolwana)	Plant extract	3 (3)		

Abbreviations: BZ, benzimidazoles; IMD, imidazothiazoles; ISN, isoquinoline; ML, macrocyclic lactones; SLD, salicylanilides.

The majority of farmers (96%) did not move their sheep to cleaner pastures after administering anthelmintic drugs (Table 5). Anthelmintic drugs were typically administered using a 5‐ or 10‐mL syringe (69%) or a drench gun (31%), with 92% of the farmers claiming to have calibrated their instruments prior to the administration of anthelmintic drugs. Most farmers (90%) had never received training on how to administer dewormers and did not estimate sheep weight before administering the anthelmintic drugs (91%). Farmers typically decided to treat their sheep either at the same time each year (47%) or when signs of worm infection appeared (37%). On more than half of the farms (60%), sheep were treated one to three times per year, and newly acquired animals were quarantined on most farms (64%) (Table 5). Additionally, none of the 107 farmers had conducted monitoring or assessment for anthelmintic efficacy. Other major helminth control malpractices encountered included the interaction of treated and untreated animals within the community (100%) and blanket treatment of animals (94%) (Table 5). Farmers (52%) regarded veterinary sales representatives as the most important advisors on helminth control issues and preferred not to discuss worm control issues with fellow farmers or community (35%) (Table 5).

**TABLE 5:** Helminth control practices on communal sheep farms within the Oliver Reginald Tambo District, South Africa.

Query	Response category	No. of response	Percentage (%)
Do you move sheep to clean pastures after treatment?	Yes	4	4
	No	103	96
Which instrument do you for oral deworming?	Drench gun	33	31
	Syringe (5 or 10 mL)	74	69
Do you calibrate the deworming instrument before use?	Yes	98	92
	No	9	8
Have you been trained to administer dewormers?	Yes	11	10
	No	96	90
Do you estimate sheep weight before deworming?	Yes	10	9
	No	97	91
How do you decide when to deworm sheep?	Same time annually	50	47
	When signs of worm infection appear	40	37
	Advice from veterinarian	3	3
	Advice from farmer	12	11
	Pre/post‐lambing	2	2
Interaction of treated and untreated sheep	Yes	107	100
	No	0	0
Do you monitor anthelmintic drug efficacy on the farm?	Yes	0	0
	No	107	100
Annual treatment frequency	<3 times	64	60
	>3 times	43	40
Do you quarantine and treat new animals for worms?	Yes	68	64
	No	39	36
Do you treat the entire sheep flock for worms	Yes	101	94
	No	6	6
Who provides you with information about worm control?	Veterinarian/animal health technician	32	30
	Sales representative/teller	56	52
	Fellow farmers	19	18
Do you usually discuss worm control practices in your community?	Yes	37	35
	No	70	65
Have you experienced anthelmintic control failure on your farm?	Yes	66	62
	No	41	38
Clinical signs that did not disappear after worm treatment (*n* = 78)^a^	Poor‐quality fleece	63	81
	Persistent diarrhoea	60	77
	Bottled jaw	59	76
	None	9	12
Anthelminthic control failure^b^	Present	48	62
	Absent	30	38

^a^ Farmers provided multiple responses to the query and faecal samples collected for detection of helminths.

^b^ Anthelmintic control failure deemed to be evident: helminth eggs detected in faecal samples on days 7 and 14 post‐treatment had an egg per gram 200 or more, and the farmer reported treatment failure with the following clinical signs present despite anthelmintic administration: bottle jaw, rough and poor‐quality fleece, and persistent diarrhoea.

The farmers noted that poor‐quality fleece (81%) persistent, diarrhoea (77%) and bottle jaw (76%) did not disappear within 3 weeks of helminth treatment. Anthelmintic control failure was deemed to be evident on 62% of the farms (Table 5). The predominant sheep crossbreed raised on the farm (Dorper‒Merino), use of 5‐ or 10‐mL syringes for anthelmintic drug administration, and the absence of sheep weight estimation were statistically associated with the reported anthelmintic control failure (*p* ≤ 0.05) (Table 6).

**TABLE 6:** Analysis of risk factors associated with reported helminth control failure on communally reared sheep farms within the Oliver Reginald Tambo District, South Africa.

Risk factor	Response category	Anthelmintic control failure reported on the farm		Chi‐square (*p*‐value)
		Yes (*n* = 48)	No (*n* = 30)	
Sheep crossbreed on the farm	Dohne‒Merino	6	10	4.914 (0.027)
	Dorper‒Merino	42	20	
Annual treatment frequency	Less than three times	33	23	0.571 (0.449)
	More than three times	15	7	
Blanket treatment	Yes	47	27	2.378 (0.123)
	No	1	3	
Absence of drench and shift approach	Yes	3	1	0.323 (0.570)
	No	45	29	
Type of instrument used to administer dewormer	Drench gun	5	10	6.242 (0.013)
	Syringe (5 or 10 mL)	43	20	
Sheep weight estimation before dosing	Yes	2	6	5.028 (0.025)
	No	46	24	

## DISCUSSION

Anthelmintic control failure is a growing challenge in resource‐constrained farming systems of South Africa.^8^ This study identified several farm attributes and worm control practices that could have contributed to the farmer‐reported anthelmintic control failure in communal sheep farms of the ORTDM of the Eastern Cape of South Africa. Livestock farming in communal areas of the ORTDM is predominantly practiced by older males (aged ≥65 years) who are typically heads of households or retirees.^6^ Physical limitations associated with advanced age may hinder the correct administration of treatments and management of deworming schedules. Furthermore, these farmers may often rely on outdated livestock health information and struggle to adopt modern animal health care practices.^8^ These elderly farmers raised sheep primarily as a form of physical exercise to stay active or as a source of secondary income rather than for commercial purposes, particularly in times of crop failure.^6^, ^31^ When sheep are kept for non‐commercial reasons, farmers may not feel the financial need to invest in proper worm control and management practices.^32^

Globally as well as in resource‐poor communal farms in the ORTDM, helminth control primarily depends on the use of synthetic anthelmintic drugs. Anthelmintic control failure is an inevitable consequence of relying solely on chemical treatments without complementary strategies such as pasture management or the use of non‐chemical control methods.^33^ Frequent and indiscriminate use of synthetic anthelmintic drugs exerts selection pressure, enabling some worms to survive exposure and reducing the efficacy of these treatments. The repeated and prolonged use of Valbantel, Maxicare and Prodose Orange, all co‐formulations of benzimidazoles and salicylanilides on these farms, may have contributed to the development of resistant worm strains.^34^ It is highly likely that the reported anthelmintic control failure resulted from inaccurate drug administration or dosing, resulting from the use of 5‐ or 10‐mL syringes for anthelmintic drug administration, and lack of sheep weight estimation. The absence of sheep weighing, is a common risky practice that has been associated with the occurrence of AR in all five districts of Limpopo^8^ and two districts of the North West^9^ provinces of South Africa. According to the sustainable worm control guidelines, sheep must be weighed and stratified by weight before deworming.^35^ Absence of weighing may result in under or over‐dosing. Under‐dosing results in insufficient drug concentration to kill all the worms, leading to anthelmintic control failure.^8^, ^36^ The use of proper instruments is critical for the effective administration of anthelmintic drugs in sheep. Nginyi et al.^24^ identified the use of syringes for drenching animals as a potential risk factor for AR in sheep and goats in coastal Kenya. The short length and pressure of a syringe deliver an anthelmintic drug into the buccal cavity. When swallowed, due to the action of the oesophageal groove, some or all of the dose may bypass the rumen and go directly to the abomasum, where it is quickly absorbed and metabolised, as a result, the helminths may not be exposed to the drug for a sufficient duration to achieve the expected level of effectiveness.^34^, ^37^ Suboptimal doses can expose helminths to sub‐lethal drug levels and uneven drug exposure, allowing resistant individuals to survive.^38^ There was a very diversity of drugs or combinations used by the farmers. Farmers who experienced treatment failure, may have responded by frequently switching drugs or combinations. Additionally, incorrect dosing or improper administration methods could have contributed to the perceived treatment failure, further driving frequent drug changes. In South Africa, anthelmintics are readily accessible at local veterinary drug shops and are often administered by farmers without veterinary supervision.^11^ Therefore, it is essential to develop and enforce policies regulating the sale and use of anthelmintic drugs in South Africa.

A farm attribute that could have contributed to the reported anthelmintic control failure was the rearing of Dorper‒Merino sheep crossbreeds. The Dorper‒Merino crossbreed is preferred for its potential in wool and meat production as well as its ability to withstand harsh environmental conditions.^39^ However, this sheep crossbreed is highly susceptible to helminth infections.^39^, ^40^ It has been reported that Dorper‒Merino crossbreeds tend to have higher worm burdens than other crossbreeds, such as Dohne‒Merino, indicating lower resistance to helminths, particularly the barber‐pole worm (*H. contortus*).^40^ In a study evaluating resistance to *H. contortus* in Sabi and Dorper sheep breeds, Matika et al.^20^ found that the Dorper breed was more susceptible to the parasite than the Sabi breed, exhibiting significantly higher faecal egg counts and lower packed red cell volume.

Other risky practices that may have contributed to the reported anthelmintic control failure on these sheep farms, and that have also been reported in several countries globally included the absence of monitoring for drug efficacy,^41^, ^42^ blanket treatment of sheep^43^ and absence of a drench and shift approach.^21^, ^44^, ^45^, ^46^, ^47^, ^48^ Periodic monitoring is crucial for managing AR and ensuring the sustainability of currently used pesticides.^35^, ^49^ If the efficacy of an anthelmintic drug is not assessed, ineffective treatments may be applied, leading to control failure and increased resistance.^50^ Therefore, strategies such as drug rotation or targeted approaches for managing control failure have become difficult to implement.^21^ Additionally, blanket treatment of sheep exposes the entire helminth population to anthelmintics, increasing the likelihood of resistant helminths surviving and reproducing.^43^ The absence of a drench and shift approach exposes animals to resistant helminths on the pastures.^48^ These practices collectively increase the need for more frequent treatments, thereby accelerating the development of resistance and eventually leading to anthelmintic control failure.

A major limitation of this study was that anthelmintic drug efficacy was not directly tested but relied solely on farmers’ perception of efficacy. It was not feasible to purchase the various types of anthelmintics used by all farmers (*n* = 78), and to conduct faecal egg count reduction tests, and collect faecal samples both before and after treatment. In general, many diseases can present with similar clinical signs, which do not necessarily indicate the involvement of helminths. Furthermore, the purposive selection of criteria for sampling participatory farmers, which excluded those with fewer than 40 sheep, potentially omitting farms where anthelmintic control failure might have been prevalent. Another limitation is the use of telephone‐administered questionnaires, which could have led to response bias due to recall errors, social desirability bias, or inaccuracies in reporting the use of anthelmintic drug use and control practices.

Given the observed statistical association between the rearing of Dorper‒Merino crossbred sheep and reported cases of anthelmintic control failure on communal farms of the ORTDM, sheep farmers should be encouraged to consider alternative breeds with greater resistance to helminth infections. Furthermore, awareness campaigns led by district veterinary services are urgently needed to educate resource‐poor farmers on sustainable helminth control practices, emphasising on the importance of weighing animals before dosing and using calibrated drench guns to ensure accurate and effective drug administration. These findings have important implications for improving parasite management in South African communal farming systems and may also provide valuable insights for similar livestock production settings across other resource‐limited regions in Africa.

## AUTHOR CONTRIBUTIONS

*Conceptualisation, methodology, data collection, sample analysis, data analysis, validation, data curation, writing—initial draft and writing—revisions*: Songezo Mavundela. *Student supervision, conceptualisation, validation, data curation, writing—revisions of manuscript and project leadership*: William Diymba Dzemo. *Student supervision, conceptualisation and validation*: Oriel Thekisoe. All authors read and approved the final manuscript.

## CONFLICTS OF INTEREST

The authors declare they have no conflicts of interest.

## ETHICS STATEMENT

To ensure compliance with ethical standards, informed consent was obtained from all participants involved. Farmers and animals were treated respectfully, and all experimental protocols used in this study were performed in accordance with appropriate guidelines that were approved by the Walter Sisulu University Research Ethics Committee, with protocol number WSU/FNS‐GREC/2024/01/11/A1.

## Supporting information



SUPPORTING INFORMATION

## Data Availability

The datasets generated during and/or analysed during the current study are available from the corresponding author upon reasonable request.
