# Supplementary material for: Anthelmintic control failure and associated risk factors reported by farmers in communal sheep farming, Oliver Tambo District, Eastern Cape, South Africa
Source: Vet Rec Open. 2025 Nov 14;12(2):e70022. doi: 10.1002/vro2.70022 (PMC12616874; doi:10.1002/vro2.70022)
Supplement: Supplementary file 1 — SUPPORTING INFORMATION [file VRO2-12-e70022-s001.pdf]

Anthelmintic control failure and associated risk factors reported by farmers in communal sheep farming, Oliver Tambo District, Eastern Cape, South Africa.

Veterinary Records

Songezo Mavundela[1], William Diymba Dzemo[1], Oriel Thekisoe[2]

[1]Departmanent of Biological and Environmental Sciences, Walter Sisulu University, Private Bag X1, Mthatha 5117, South Africa.

[2]Unit for Environmental Sciences and Management, North-West University, Potchefstroom 2531, South Africa.

***Corresponding author email:** wdzemo@wsu.ac.za

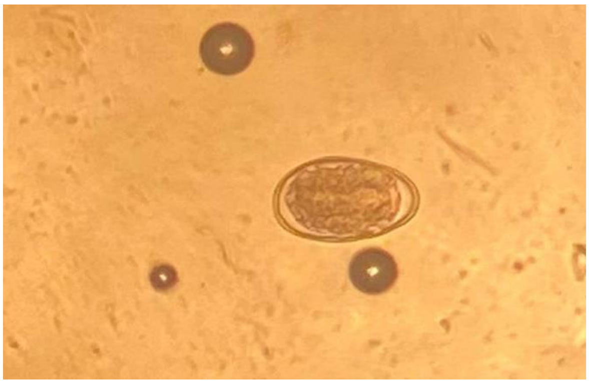

A. *Strongyloides* spp. egg

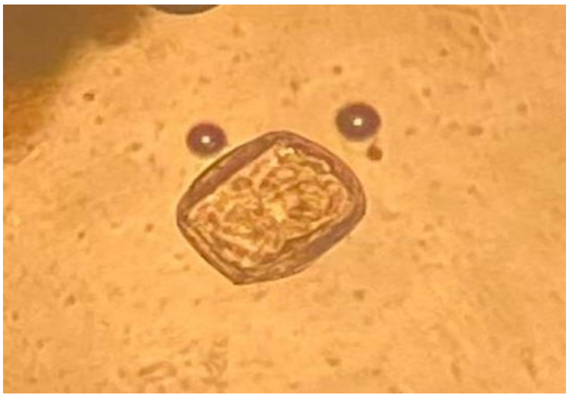

B. *Moniezia* spp. egg

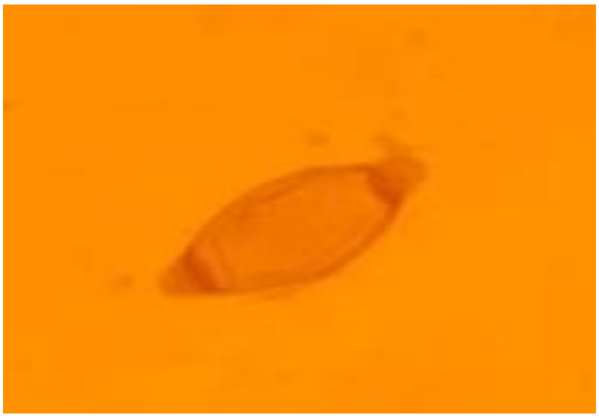

C. *Trichuris* spp. egg

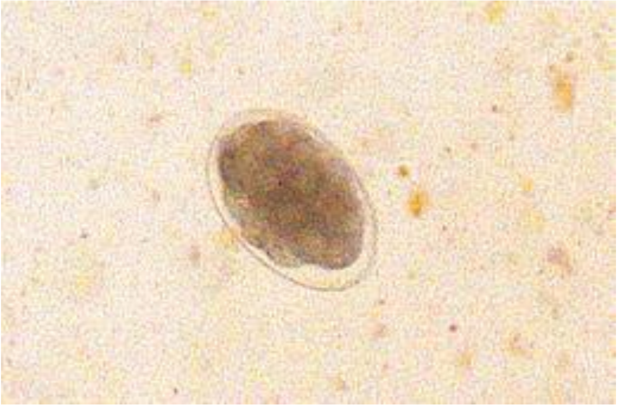

D. *Haemonchus* spp. egg

Supplementary Figure 1 Micrographs of helminth eggs identified from faecal samples collected from sheep on communal farms within the Oliver Reginald Tambo District Municipality of South Africa.
